# Pain during Rapid Maxillary Expansion: A Systematic Review

**DOI:** 10.3390/children10040666

**Published:** 2023-03-31

**Authors:** Martina Barone, Alberto De Stefani, Filippo Cavallari, Antonio Gracco, Giovanni Bruno

**Affiliations:** 1 Department of Neuroscience, School of Dentistry, University of Padova, 35122 Padova, Italy; 2 Department of Pharmacological Sciences, University of Padova, 35122 Padova, Italy; 3 Azienda Ospedaliera di Padova, 35122 Padova, Italy; 4 Department of Industrial Engineering, University of Roma Tor Vergata, 00133 Roma, Italy

**Keywords:** pain, RME (rapid maxillary expansion/expander), RPE (rapid palatal expansion/expander)

## Abstract

Aim: The aim of the present systematic review is to evaluate the pain perceived by patients during rapid maxillary expansion (RME) in relation to factors such as demographic characteristics, appliance type, activation protocol, and the eventual use of medication or pain management strategies. Materials and methods: An electronic search of available articles on the subject was conducted on three electronic databases, using predefined keywords. Sequential screenings based on pre-established eligibility criteria were performed. Results: Ten studies were ultimately included in this systematic review. The main data of the reviewed studies were extracted according to the PICOS approach. Conclusions: Pain is a common effect of RME treatment that tends to decrease over time. Gender and age differences in pain perception are not clear. Perceived pain is influenced by the expander design and expansion protocol used. Some pain management strategies can be useful for reducing RME-associated pain.

## 1. Background

Rapid maxillary expansion (RME) is an orthodontic–orthopedic treatment routinely performed worldwide in growing subjects with transversal deficiency of the maxillary arch, with or without a crossbite [1,2,3,4]. This treatment can be performed in primary, mixed, or permanent dentition [1,5,6]. 

According to Baccetti et al. [7], while an RME performed before the peak of pubertal growth leads to significant and more effective long-term skeletal changes in maxillary and circum-maxillary structures, the same treatment performed after the peak tends to have greater effects at the dentoalveolar level than at the skeletal one. This is due to the different mid-palatal suture maturation stages that can be observed in various age ranges: in the ‘‘infantile’’ stage (<10 years), the suture is broad and smooth; in the ‘‘juvenile’’ stage (10–13 years), it appears with a more typical squamous aspect, with overlapping sections; and in the ‘‘adolescent’’ stage (13–14 years), the suture is wavier, with increased interdigitation [7,8].

The type of appliance used for RME treatment can include a dental, skeletal, or dento-skeletal anchorage. However, the rationale for the appliance is always the same: to separate the mid-palatal suture [9,10,11]. Therefore, RME requires the use of heavy dentofacial orthopedic forces, to produce skeletal effects by minimizing the unwanted dental ones, such as molar tipping and alveolar bending [1,10]. The expansion protocol chosen by the clinician generally prescribes one to three activations per day, for a period ranging from approximately two to four weeks [1,10].

Patients often report having pain or discomfort during the active phase of RME [3,5], especially during the first activations. The literature suggests that pain is the most commonly reported symptom, with a frequency of >90% among children [2,12].

The purpose of this systematic review of the literature is to evaluate the pain perception reported by patients during RME in relation to factors such as age, gender, appliance type, activation protocol, and the eventual use of medication or pain control strategies.

## 2. Materials and Methods

The PRISMA (preferred reporting items for systematic reviews and meta-analyses) guidelines [13,14] were followed for the present systematic review of the existing literature. This review protocol was registered on the International Prospective Register of Systematic Reviews (PROSPERO; registration number: CRD42023403349).

The focused PICOS question pursued by the authors in this systematic literature review was: “Is the pain experienced by patients during rapid maxillary expansion (RME) related to factors such as demographic characteristics, appliance design, activation protocol, and/or the use of any medication or strategy to control the pain?”.

A search of the electronic databases PubMed, Scopus, and Web of Science was conducted in October 2022. The keywords used by the researchers were: “pain,” “rapid palatal expansion,” and “rapid maxillary expansion.” The same keywords were used in all three of the databases, and no filters were set.

### 2.1. Selection of Studies

After the removal of duplicate results, the study selection phase was performed based on pre-established eligibility criteria. 

Automation tools were not used for the study selection process. Instead, two authors independently performed an accurate analysis of the titles and abstracts of the articles that emerged from the research on the electronic databases. To calibrate inter-examiner reproducibility, the following method was used: in case of disagreement regarding the inclusion of a study, the two authors discussed and reached a mutual consensus before coming to a final decision. 

The selection process was based on the type of article (publications as reviews, meta-analysis, letters, comments, case reports/series, surveys not on human were not included), the language (only articles in English were included), the age of the study sample (articles in which the study sample presented a mean age higher than 13 years or in which an age range over 13 years was evaluated were excluded), the availability of the abstract and/or the full-text article (if those were not available, the study was excluded), and the article’s relevance to the aim of the present systematic review (the studies whose aims were not relevant to those of the review were excluded). For articles that met a combination of inclusion and exclusion criteria, a full-text analysis was performed before selecting those to be included and reviewed in this investigation.

### 2.2. Data Extraction 

The characteristics and main data of the included studies were extracted according to the PICOS approach:

*P* (patients/problem/population): patients aged up to 13 years being treated with rapid maxillary expansion.

*I* (intervention): rapid maxillary expansion.

*C* (comparison): if a comparison was done.

*O* (outcome): pain. 

*S* (study design): randomized clinical trials and observational analytical studies, though only studies with human participants.

Relevant data from each included article were collected and organized in a table (Microsoft^®^ Office 365^®^ Excel). No automation tools were used for the data collection process.

### 2.3. Study Quality Assessment

The articles included in the full-text analysis were evaluated for their quality based on methodological quality criteria adapted from the CONSORT statement, the Jadad quality assessment scale, and previous studies (Table 1) [4].

Two reviewers independently scored each study; disagreement in the scoring was solved with discussion and, if necessary, consultation with a third author. The total possible score for each article was 11 points, and studies were classified as follows: good, with a total of >9 points; moderate, with 7–9 points; and poor, with <7 points.

## 3. Results

### 3.1. Study Selection

The research conducted on the PubMed, Scopus, and Web of Science databases led to a total of 265 articles (83 on PubMed, 97 on Scopus, and 85 on Web of Science). After the process of removing duplicates, a title and abstract analysis was performed for 136 articles. This process led to the exclusion of another 126 articles. Ten studies were included in the full-text analysis, and all of them were then included in the final systematic review.

**Figure 1:**
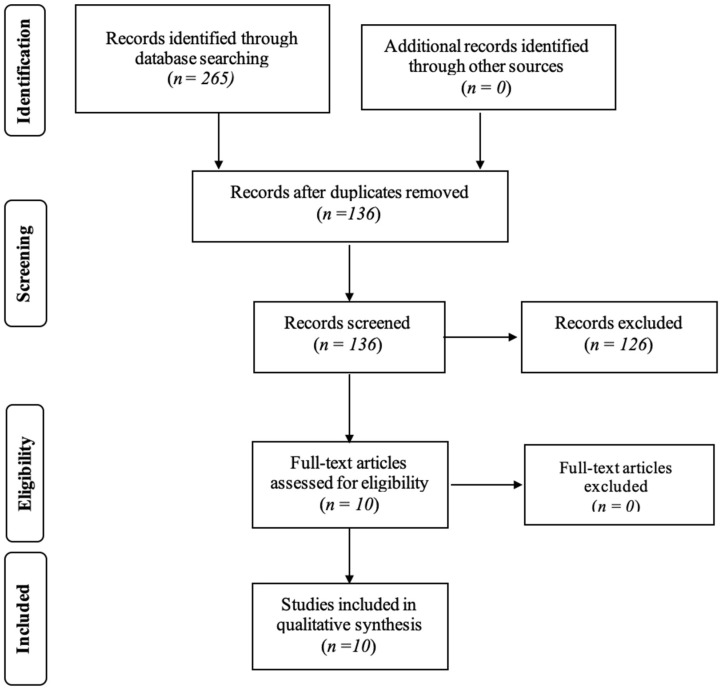
Article screening: four-phase PRISMA (preferred reporting items for systematic reviews and meta-analyses) flow diagram for study collection, showing the number of studies identified, screened, deemed eligible, and included in the present review.

Figure 1 shows the study selection phase. The figure describes the number of studies that were identified, screened, deemed eligible, and ultimately included in the present review.

### 3.2. Study Characteristics

Table 2 and Table 3 summarize the main data collected from analysis of the studies included in the present systematic review. It must be specified that, for the aim of the present review, only the portion of the included studies concerning RME-associated pain and the relevant reported data were considered. These extracted data are reported in Table 1.

The most recent study included was published in 2022 by Caccianiga et al. [2], while the oldest one was published in 2000 by Needleman et al. [10]. Regarding study design, five studies were randomized clinical trials [2,12,15,16,17], one was a prospective study [3], one was a parallel cohort study [1], and one was divided into two phases, the first of which was a randomized, controlled clinical trial and the second of which was a prospective case series (The authors decided to include the second part of this study in the review too because, although it was defined as a prospective case series, a statistical analysis of the data was still performed) [9]. For two studies, the study design was not clearly defined by the authors [5,10].

Pain level was assessed in the included studies using different scales. Some studies used a single scale, while others utilized two scales to measure pain in their study sample. The scales used were of five types:

The Wong–Baker Faces Pain Scale (FPS) was used in five studies [1,3,10,12,15], while one study utilized a revised FPS [5].

The Numerical Rating Scale (NRS) was used in two studies [2,3].

The Visual Analogue Scale (VAS) was used in three studies [9,16,17].

The Graphic Rating Scale (GRS) was used in one study [1].

The Color Analog Scale (CAS) was used in one study [10]. 

In the majority of the included studies, patients registered their pain daily after each activation of the appliance during the active phase of the maxillary expansion, according to the activation protocol used. 

### 3.3. Quality Assessment of the Selected Studies 

Table 4 shows the evaluation of the methodological quality of the studies included in this systematic review. 

With regard to the studies’ methodological quality, four studies were classified as good [9,12,15,16], four as moderate [1,2,3,17], and two as poor [5,10].

## 4. Discussion

Rapid maxillary expansion is a procedure that is widely and routinely performed in cases of maxillary contraction, with the aim to correct transverse discrepancy and normalize the transverse relationship between the upper and lower dental arches. A common side effect is the pain associated with the active expansion phase, during which one or more appliance screw activations are performed in order to open the mid-palatal suture. 

The present systematic review of the literature aims to evaluate the pain perceived during rapid maxillary expansion in relation to age, gender, appliance type, activation protocol, and the eventual strategies of pain management used. 

In all of the included studies, patients reported some pain. The pain appeared to be higher during the first days of treatment (usually within the first weeks) or the first screw activations, decreasing as treatment progressed. This data is in agreement with the literature [6,11,16,18]. 

### 4.1. Pain and Gender

Not all of the included studies provided information about the difference in pain perception during RME between the two sexes. Some studies [5,10,15] described the absence of a statistically significant difference between the two sexes in reported pain. However, de Araújo et al. [3] reported higher pain levels in females than in males during RME treatment, while Feldman et al. [17] reported that females complained of more dental tension than males, even if with minimal differences. Cesur et al. [5] focused on the level of pain at different dental arch sectors, and reported significantly lower pain levels in males in the posterior teeth area on the sixth day of expansion compared to the first and second days. 

These findings reflect the existing literature, there is an absence of agreement on whether pain perception may or may not be influenced by gender. While some studies report the absence of a difference between the sexes, some others report that females appear to be more sensitive to pain [6,11,16,18].

### 4.2. Pain and Age

The authors of this article decided to exclude from the review studies that had sample populations with a mean age or age range higher than 13 years old. The aim of this study is to evaluate pain perception during RME only in children and pre-teens, since this population constitutes the age at which this procedure is more frequently executed. Since pain perception could be different in adults versus children/pre-teens, these exclusion criteria reduce the possible bias a wider age range could introduce. 

Not all of the included studies provided information about the difference in pain perception at different ages. Needleman et al. described the absence of a statistically significant difference in reported pain according to patient age, or between expansion rate and age. According to the study by Feldman et al., age was positively correlated with overall pain and discomfort on the fourth day of treatment. In the study by Matos et al. it was observed that younger patients tended to experience less pain; specifically, 7-year-old children experienced less pain than 11-year-olds. 

Therefore, based on the literature [16], it is not clear whether or not age influences the perception of pain. 

### 4.3. Pain and Type of Expander Used

Some studies compared the influence of different RME appliance types on pain perception reported by patients. 

De Araujo et al. [3] compared two types of traditional maxillary expanders, Hyrax and Haas appliances, and reported significantly higher pain after 1 day of therapy in patients treated with the Hyrax expander. However, with the exception of the first treatment day, the authors concluded that the type of appliance used did not significantly influence the perception of pain consequent to RME. The expander design could explain the higher pain level reported during the first day of activation by the patients treated with the Hyrax appliance. In fact, the two types of expanders are both anchored to the tooth with cemented bands, but in the Hyrax appliance the screw is connected to the bands only with a rigid stainless-steel structure, while in the Haas appliance there are also two acrylic pads connected to the stainless-steel structure and rested on the palate. Thus, if the Hyrax transmits the forces only to the periodontium of the supporting teeth, the Haas distributes them to the palate and buccal bone plate areas too [3,19]. 

Two included studies compared tooth-borne with tooth–bone/bone-borne RME appliances. Feldmann et al. [17] did not find statistically significant differences between the two groups in terms of pain, although patients with tooth-borne RME appliances generally reported higher pain levels than those with tooth–bone-borne RME appliances. In the opinion of the authors of the study, this could be explained by the fact that the center of the force generated by the screw activation is closer to the mid-palatal suture in tooth–bone-borne RME appliances, because of the skeletal anchorage, so the quantity of force distributed to the dentition could be reduced and attenuated, resulting in less pain experienced by the patient. Conversely, in the study by Altieri et al. [1], patients with bone-borne RME devices reported statistically significant higher pain levels during the first day of activation compared to those with tooth-borne RME expanders. They did not show statistically significant differences on subsequent days, even if patients with bone-borne RME appliances generally reported higher pain levels. According to the authors, the pain during the first day could have been partly caused by the insertion of miniscrews. 

Therefore, in the studies included in the present review there was not a clear agreement on the role of anchorage type (i.e., dental vs skeletal/dento-skeletal) in pain associated with RME. 

Two of the included studies [12,16] compared the traditional RME appliance with the Leaf expander (LE), which is made up of a shape memory double nickel–titanium leaf spring. The rationale of the shape memory leaf spring is to apply continuous force that, along with the superelastic property of the nickel–titanium, leads to a more physiologic, calibrated, and comfortable expansion for the patient with the LE compared to one with a traditional RME expander [12,20,21]. The forces transmitted to the bone and sutural complex appear decreased thanks to the slow and continuous activation performed by the LE, causing a consequent inhibition of the tissues’ inflammatory response, which clinically results in reduced pain perception for the patient during the appliance activation [12,22]. Both of the studies reported a statistically significant decrease in pain in patients treated with LE, in the first 4 days according to Ugolini et al. and in the first week according to Nieri et al. Thereafter, pain tended to decrease in both groups in both studies, without other significant differences. However, Nieri et al. considered the difference between the LE and RME probably not clinically significant, since the difference in VAS score between the two studied groups was very small (i.e., 0.3 points).

### 4.4. Pain and Activation Protocol Used

Different activation protocols were used by authors in the included studies. For each study, the activation protocol described in Table 2 was maintained during the entire period of investigation. In all of the included studies, a certain level of pain was reported by patients, which is why it appeared difficult to define a relationship between pain reported and activation protocol used. In the study by Needleman et al., some patients were prescribed 1 activation/day while others were prescribed 2 activations/day, based on the individual preference of the treating orthodontist. It emerged that patients whose activation protocol was 2 activations/day were 2.1 times more likely to report pain than patients with an activation protocol of 1 activation/day (three times more for the first 10 activations). This finding accords with the literature; the type of activation protocol can influence the pain perceived during RME. Specifically, a slower activation protocol (i.e., less screw activation per day) is correlated with a lower pain level for the patient [6,11,23].

### 4.5. Pain and Strategy of Pain Management Used

In two of the included studies [2,15] the effect of photobiomodulation therapy (PBMT) on pain occurring during/after RME was evaluated. For the purpose of these studies, a control group not receiving PBMT was enrolled. PBMT uses low-powered laser light within the red-to-near-infrared range to achieve biological responses. Its likely capacity for orthodontic pain reduction is due to the inhibition of arachidonic acid release, with a consequent decrease of prostaglandin E levels, and to the induction of beta-endorphin release, which causes an efficient analgesic reaction [2]. The two studies had a similar sample size (34 [15] vs. 30 [2] patients), but the mean age of the sample was slightly lower in the Caccianiga et al. study. The type of laser used, as well as the protocol of irradiation, were different between the two studies: Caccianiga et al. utilized a laser for extraoral irradiation and only performed this on the day of expander positioning. Matos et al. performed the irradiation with an intraoral laser in the mid-palatal suture area, and patients received four irradiations during the active expansion phase (the first one at the expander positioning) and eight more irradiations after screw fixation (one per week for 8 weeks). 

Caccianiga et al. found the laser to be efficient for alleviating pain intensity and reducing pain duration during the active RME phase. Pain scores reported by the patients were always significantly lower in the laser group at each detection than in the non-laser group. Conversely, Matos et al. reported a greater risk of experiencing high pain levels in the laser group than in the control group. It must be mentioned that in this study the control group was exposed to placebo irradiation; it is natural to wonder, therefore, if the placebo effect could have effected a greater action in terms of pain perception than the laser itself. However, this data was not statistically significant, and the authors of the study concluded that the laser had no effects in terms of painful sensation alleviation during the active phase of expansion.

It might come as a surprise that the study in which a statistically significant result was obtained for the management of RME-associated pain is the one by Caccianiga et al. In this study, the irradiation did not occur directly near the area of the mid-palatal suture (where the expander exerts its action), as instead an extraoral PBMT laser was used. These patients received the irradiation only once, on the day of appliance application (so even before the beginning of the active phase of expansion). Nevertheless, as explained by Caccianiga et al., the laser they used (ATP38^®^) allowed the simultaneous irradiation of all the circummaxillary sutures; this may have allowed the laser to exert a greater effect on the overall manifestation of pain, compared to a device acting only at the level of the mid-palatal suture.

Despite the fact that PBMT is a technology with various different applications in medicine and dentistry today [2,24,25], it is not available in all dental clinics, whether public or private. Therefore, even if it is useful to know its effects on RME-associated pain alleviation, PBMT cannot be considered a routine tool for this purpose. However, since it has no side effects, dental clinics that already use PBMT may find it useful for relieving pain in patients starting RME therapy.

With regard to pharmacological pain management, in some included studies the use of analgesic and/or anti-inflammatory drugs during the observation period was completely prohibited [2], prohibited without prescription [1], or allowed at one’s discretion (i.e., without indicating the type of medication or regimen) [3,12,17]. In the study by de Araújo et al., in which patients were treated with Hyrax- and Haas-type appliances, no analgesic use was declared, although 100% of them reported some pain during the expansion period. Ugolini et al. reported that the 25.2% of patients treated with traditional RME devices used analgesics, compared to 0% of patients treated with LEs. In the study by Feldmann et al., a low use of analgesics was reported by the patients of both studied groups (i.e., tooth-borne expanders and tooth–bone-borne expanders), without statistically significant differences between them; the most used analgesics by patients in this study were Paracetamol and Ibuprofen.

Needleman et al. reported how the 48% of studied patients used drugs at least once during the expansion phase, with no differences based on age or gender. Drugs were taken after 7% of activations and 69% of the time during the first six screw activations; no differences were reported during the last 10 days of appliance activation. The reported drugs were Tylenol^®^ (i.e., Paracetamol), Advil^®^ (i.e., Ibuprofen), and Motrin^®^ (i.e., Ibuprofen).

The study by Cossellu et al. [9] was the only one which specifically analyzed the effects of the use of analgesic drugs on pain management during RME. In its first phase, the study compared the effect of 40 mg ketoprofen lysine salt (KLS) to that of 250 mg paracetamol/acetaminophen (P), by evaluating which of the two drugs was the most effective in pain reduction. Furthermore, the study evaluated whether the use of an analgesic during the first 3 days of appliance activation could be effective in significantly reducing pain from the first treatment day. Patients did not receive a pharmacological regimen prescription to follow: they were only asked to report if and on what day/s they used the indicated analgesics.

KLS appeared to be more effective than P, probably due to its anti-inflammatory as well as analgesic properties. In fact, KLS belongs to the class of drugs defined as NSAIDs, which block COX-1 and/or COX-2, whereas P blocks COX-3, which is only expressed in the brain and spinal cord. Therefore, while KLS acts across cell membranes and is able to produce effects that reduce local synthesis of PGs (i.e., molecules that enhance the transmission of painful stimuli and increase sensitivity to noxious stimuli), P acts only on the central nervous system, with minimal peripheral consequences [26,27,28]. Thus, KLS appears to be more effective for inhibiting the acute inflammation response caused by the vasodilation resulting from the orthopedic forces exerted by the maxillary expander [29].

The second phase of this study by Cossellu et al. showed that the use of KLS during the first 3 days of screw activation seemed to be even more effective at reducing pain from the first day, with patients reporting almost no pain for the whole active phase of RME. It should be mentioned that, as stated by the study authors themselves, patients treated with RME can have different perceptions of pain. Therefore, it’s possible that in this phase of the study, the KLS group might have been composed of patients who otherwise would not have needed analgesic—which could constitute a bias. Nevertheless, as all patients in the first phase of the study reported pain and the necessity for a medication to manage it, the authors believed that an analgesic should be suggested during the first day of RME.

### 4.6. Limitation and Future Suggestions 

The sensation of pain is complex; pain perception, as well as individual tolerance level, can vary between different subjects [6]. It therefore appears difficult to objectively quantify pain. A limit of the present systematic review is that in all the included studies pain was subjectively evaluated using questionnaires, thus only the pain as reported by patients was considered; it was not measured with any dedicated, appropriate device. In fact, individual levels of pain perception and tolerance can cause subjects with the same objective degree of pain to subjectively declare different levels of pain on the same pain scale. For a more precise and objective pain assessment and to reduce the risk of subjective-perception-related bias, studies in which pain assessment is objectively performed with proper devices would be needed. However, it appears that this type of study could be very difficult to achieve. 

Another limitation of this review is that none of the included studies evaluating the use of analgesics for the management of RME-associated pain indicated a clear pharmacological regimen to follow. Future studies evaluating the efficacy of different types of analgesic and/or anti-inflammatory drugs, with a regimen defined by clinicians, would be interesting and useful for identifying the most effective drug types and respective dosages for controlling RME-associated pain. Such a study might also be able to define a standardized pharmacological protocol that all orthodontists could prescribe to their patients.

## 5. Conclusions

Pain is a common effect of RME treatment, which tends to occur during the first few days/activations and then decrease over time.

No clear difference in perceived pain was observed between different ages or genders.

Expander design may affect perceived pain: Haas and LE appliances appear to result in a lower patient pain levels, though there is no clear agreement about appliances with dental vs skeletal/dentoskeletal anchorage.

A slower expansion protocol (i.e., fewer screw activations per day) correlates with less pain perceived by the patient.

Extraoral PBMT can be a useful adjunct in the management of RME-associated pain.

KLS seems to be the most valid drug option for reducing and preventing RME-associated pain, thanks to its analgesic and anti-inflammatory properties.

## Figures and Tables

**Table 1:** Methodological quality criteria.

Sr No.	Items	Scoring
A	Design of randomized clinical trial	1
B	Eligibility criteria for study participants	1
C	Sample size determination	1
D	Details about clinical diagnostic criteria	1
E	Ethical considerations	1
F	Method of blinding	1
G	Methods and type of randomization	1
H	Description of recruitment period and follow-up	1
I	Withdrawals and dropouts	1
J	Clearly defined outcomes	1
K	Appropriate statistical analysis	1
	Total score	11

**Table 2:** Characteristics and main data of the reviewed studies, extracted according to the PICOS approach. Part 1.

Author/Year	Patients/Problem/ Population	Intervention
Caccianiga et al., 2022 [2]	30 patients (15 PBMT G, 15 no PBMT G). 16 F, 14 M. M.a. 7.8 years (7.6 years PBMT G, 8 no PBMT G).	- To demonstrate the effect of PBMT on pain after RME in growing subjects. - Activation protocol: 2 activations/d, 12 h apart, for 7 d (^2^/_4_ turn/d, 0.5 mm/d). - PBMT G: received extraoral irradiation (in three consecutive cycles) at the RME positioning site, using an ATP38^®^ laser. - Pain level: measured with NRS (0–10 pt) at 6, 12, 24 h, and after each d, up to the 7th d post RME positioning. - No analgesic/anti-inflammatory drugs were used during the active phase.
de Araújo et al., 2021 [3]	39 patients with posterior crossbite or maxillary atresia: 20 Hyrax G, 19 Haas G. M.a.: 9.35 years (9.56 Hyrax G, 9.13 Haas G). Hyrax G: 56.5% F, 43.7% M. Haas G: 43.5% F, 56.3% M.	- To compare the intensity of pain caused by RME using Haas- vs. Hyrax-type appliances during the growth stage. - Activation protocol: initial activation of one full turn on the 1st d, followed by ^2^/_4_ of a turn/d until a screw opening of 7 mm was reached. - Pain level: measured 15 min after each turn, using a combination of NRS and Wong-Baker FPS pain scales.
Matos et al., 2021 [15]	34 patients (18 PBMT G, 16 no PMBT G). PMBT G: 45.5% F, 55.5% M. M.a. 9.2 years. No PBMT G: 55.6% F, 44.4% M. M.a.8.2 years.	- To assess the influence of PBMT on mid-palatal suture bone formation and pain sensation of patients treated with RME (Hyrax type). - Activation protocol: one full turn at the time of RME application, followed by two daily ^1^/_4_ turns (at an interval of 12 h) from the 2nd d after insertion, until the lingual cusps of the upper posterior teeth occluded with the buccal cusps of the lower posterior teeth. - PBMT G: received PBMT in the mid-palatal suture area (four irradiations during the active phase and eight more after screw fixation); members of No PBMT G received sham PBMT. - Pain level: measured daily with a visual analog scale based on Wong-Baker FPS during the first 14 d of treatment (active phase).
Nieri et al., 2021 [16]	56 patients (28 LE G, 28 RME G) equally divided in two Italian centers. LE G: 61% F, 39% M. M.a. 8 years. RME G: 43% F, 57% M. M.a. 8.4 years.	- To compare the effects of the LE screw versus the conventional RME screw on patient-reported outcomes measured during the first 12 w of treatment. - LE activation protocol: with a Ni–Ti screw developing continuous force; initial expansion of 4.5 mm in about 2–3 m, followed by ten ^1^/_4_ turns/m for spring reactivation (1 mm). - RME activation protocol: ^1^/_4_ turn/d (0.2 mm) until the desired expansion was achieved. - Desired expansion for both Gs (activations stop): when the palatal cusps of the upper second deciduous molars approximated the buccal cusps of the lower second deciduous molars. - Pain level: measured with VAS scale (0–10 pt) once per w for 12 w.
Altieri et al., 2020 [1]	38 patients (18 TBE G, BBE G) 44% F, 56% M. M.a. 12.3 years.	- To investigate and compare the perceived pain intensity during the activation phase of RME with TBE and BBE. - Activation protocol: four ^1^/_4_ turns on the 1st d and three ^1^/_4_ turns/d in the active phase of treatment (0.20 mm per turn, 0.6 mm daily) until screw opening reached 8 mm. - Pain level: measured with GRS and Wong-Baker FPS every d, 15 min after the screw activation. - Painkillers were forbidden during the active phase in the absence of a prescription.
Ugolini et al., 2019 [12]	101 patients (48 RME G, 53 LE G). RME G: 26 F, 23 M. M.a. 9.4 years. LE G: 28 F, 25 M. M.a. 9.1. years.	- To investigate and analyze pain perception during the first w of activation with two palatal expansion screws and to identify the effect of different expansion protocols on pain perception in young patients. - RME G activation protocol: screw turned two times at chairside, followed by two ^1^/_4_ turns/d (1 in the morning and 1 in the evening, 0.40 mm/d), until over-correction. - LE G activation protocol: screw pre-activated in the laboratory for the first 3 mm of expansion, followed by reactivation performed in the office by ten ^1^/_4_ turns/m (1 mm each) until expansion completion. - Pain level: measured with Wong-Baker FPS (0-10) from the 1st to the 7th d of screw activation, with a double registration/d (morning and evening).
Cesur et al., 2018 [5]	62 patients (32 F, 30 M). F m.a.: 13.16 years. M m.a.: 12.91 years.	- To investigate the time at which pain started after RME application, the duration and intensity of pain, the teeth affected, and the importance of sex in pain perception. - Activation protocol: ^1^/_4_ turn twice/d (morning and evening) until the palatal cusps of the maxillary first molars contacted the buccal cusps of the mandibular first molars. The first activation was performed in the office. - Pain level: rated with FPS-R after each turn. After 1 w, patients completed a questionnaire in which question 1 was about the time at which pain was first perceived following the first activation. Over the following week, patients were asked each d in the morning and evening if and in which teeth they felt pain.
Cossellu et al., 2018 [9]	Phase 1: 101 patients. KLS G: 28 patients (17 F, 11 M), m.a. 8.5 ± 1.8 years. P G: 35 patients (17 F, 18 M), m.a. 8.7 ± 1.8 years. CTRL G: 35 patients (17 F, 19 M), m.a. 8.9 ± 1.2 years. Phase 2: added KLS-B 31 patients (15 F, 16 M), m.a. 8.7 ± 1.6 years.	- To compare the effects of KLS vs P on pain perception during RME. Two phases: (1) To understand which of the two analgesics is the more effective for pain reduction; (2) To test if the use of an analgesic during the first 3 d might significantly reduce pain following the 1st d of RME activation. - Activation protocol for KLS G and P G, phase 1: two turns/d (0.5 mm/d, for at least 7 consecutive d) until the occlusal aspect of the maxillary lingual cusp of the upper first molars contacted the occlusal aspect of the vestibular cusp of the mandibular first molars. To relieve pain: 40 mg ketoprofen was prescribed to KLS G, while 250 mg paracetamol was prescribed to P G during screw activation. - Activation protocol for CTRL G: same as previous, but the screw was not activated for the 1st w of treatment. - Pain level assessment in phase 1: VAS associated with a numeric rating scale, by completing a questionnaire before appliance insertion and every following d after RME. Patients reported if and when they consumed analgesic drugs. - Activation protocol phase 2: same as phase 1. Patients took analgesics once per d for the first 3 d of activation.
Feldmann et al., 2017 [17]	50 patients (25 TBE G, 25 TBBE G). M.a.: 9.7 years TBE G, 10 years TBBE G.	- To evaluate and compare perceived pain intensity and discomfort during the 1st w with TBE or TBBE. - Activation protocol: two ^1^/_4_ turns/d (0.5 mm) until the palatal cusps of the maxillary first molars contacted the buccal cusps of the mandibular first molars. - Patients were advised to use nonprescription analgesics at their own discretion. - Pain level: measured with self-report questions concerning pain intensity, discomfort, and analgesic consumption, issued on the 1st and 4th d of treatment. Questions 1–9 graded with VAS; question 10 had a binary “yes/no” response with follow-up questions. - Three questions for the 1st d in treatment concerned patients’ experiences of pain and discomfort during the appliance placement.
Needleman et al., 2000 [10]	97 patients: 61% F, 39% M. M.a.: 7.7 years.	- To investigate the prevalence, timing, and intensity of pain during RME in children and to ascertain the association between pain and age, gender, and expansion rate. - Activation protocol: One or two activations/d, based on individual provider preference. - Pain level: assessed with FPS and CAS after each activation.

F = female/s, M = male/s, m.a. = mean age, G = group, RME = rapid palatal expansion/expander, LE = Leaf expander, min = minutes, h = hour/s, d = day/s, w = week/s, m = month/s, pt = point/s, NRS = Numerical Rating Scale, FPS = Faces Pain Scale, FPS-R = Faces Pain Scale-Revised, GRS = Graphic Rating Scale, CAS = Color Analog Scale, PBMT = photobiomodulation therapy, TBE = tooth-borne expander, BBE = bone-borne expander, TBBE = tooth–bone-borne expander, KLS = ketoprofen lysine salt, P = paracetamol/acetaminophen, CTR = control.

**Table 3:** Characteristics and main data of the reviewed studies, extracted according to the PICOS approach. Part 2.

Comparison	Outcome	Study Design
PBMT G with no PBMT G.	- PBMT G: the highest perception of pain was observed at 6 h after RME application (intermediate scoring: 2), then it progressively decreased to an intermediate scoring of 1 after 12 h, and 0 after d 2. - No PBMT G: the highest perception of pain was observed at 6 and 12 h after RME application (intermediate scoring: 4), then it declined to a median of 3 after 24 h. It increased again to a median of 4 at d 3, decreased to 2 on d 4, to 3 on d 5, to 2 on d 6, and to 1 on d 7. - The pain perceived in each recorded moment and the highest pain scored significantly differed between the two Gs: considerably lower scores were observed in PBMT G. - PBMT with ATP38® laser alleviates the intensity and duration of pain perceived by few patients during RME.	Randomized clinical trial.
Hyrax G with Haas G.	- A unanimous 100% of patients reported some pain during the expansion period. - There was a statistically significant inverse correlation between pain and the number of d from RME insertion. Pain intensity was higher on the 1st d of activation. Pain was moderate or strong on the first 2 or 3 d of activation and decreased with time. - Hyrax G: reported significantly greater pain than Haas G on the 1st d. Device type did not significantly influence perceived pain (except for on the 1st d). - A greater level of pain was observed in F throughout the treatment. A total of 43.47% of F and 18.75% of M reported “the worst pain” at least once during the activation period. - Use of analgesics during the active expansion phase was not reported.	Prospective study.
PBMT G with no PBMT G.	- No statistically significant difference was observed in the risk of presenting higher levels of pain during the active phase of treatment between the two G. - Pain was significantly higher for the first 7 d of treatment than it was on the 14th d. - No statistically significant difference was observed between the sexes. - Younger patients tended to have less sensitivity to pain, with 7 year-old children reporting less pain than 11 year-old children. - A decrease in pain level was observed after a peak in both G from the 1st to the 2nd d of treatment. - PBMT had no effect on pain sensation during the active phase of RME.	Two-arm parallel-group randomized clinical trial.
LE G with RME G.	- A total of 79% of patients in LE G and 86% of patients in RME G reported pain. - The difference in pain level between the two groups was significantly different in the first w, with the LE G reporting less pain. This difference has clinical relevance. - In the 2nd w, the significant difference in pain persisted in center 2, probably due to differences in pain perception among patients. - Following w: no significant differences between the two G, the two centers and in the interaction group/center. Pain decreased progressively over the 12 w after the treatment started. - Pain was lower in LE G, with a statistically significant, but probably not clinically significant, as the difference in VAS was very small (0.3 points).	Multicenter randomized controlled trial.
TBE G with BBE G.	- Statistically significantly higher pain was observed in BBE G only on the 1st d. - No significant differences in pain levels between groups were observed for the following d, although subjects with BBE generally scored higher mean pain levels.	Parallel cohort study.
LE G with RME G.	- RME G patients (88.6%) suffered from a significant generalized pain during the first w of screw activation compared to those in LE G (25%). - A total of 25.2% of RME G patients reported analgesic consumption. - RME G reported statistically significant heightened pain levels in the first 4 d of treatment, with 51.4% of patients suffering at least one time from strong pain in the first 4 d. - LE G reported statistically lower pain, with 9.7% of patients suffering from strong pain limited to the first 2 d after cementation/activation. A total of 90% reported not perceiving any pain in the first 2 d. - From the 5th to the 7th d, only small amounts of pain were reported, with no significant difference between the two groups. - Continuous force from the Ni-Ti spring allowed patients to avoid the worst levels of pain in the first 7 d of activation. The LE appliance is effective and efficient for pain prevention.	Multicentric randomized study.
None.	- A total of 66.12% of patients reported pain after the first activation, while 87.80% perceived pain in the first 2 h. - No statistically significant sex difference was observed in patients’ pain reporting. - The percentage of patients reporting pain in the posterior teeth was greater than those reporting pain in the anterior teeth. - In the anterior teeth, no statistically significant difference was observed between the mean FPS-R scores reported each morning and evening by F vs. M. - In the posterior teeth, statistically significant results were observed between mean FPS-R scores on d 2 and 6 in the morning and on d 1 and 6 in the evening for M.	Not defined by authors.
- Phase 1: KLS G with P G and CTRL G. - Phase 2: KLS G with KLS-B G.	Phase 1: - Average pain perception over time was higher during the first 3 d (mild to moderate pain). - KLS G reported a single use of drugs during the 2nd d. Of P G, 60% reported use of drugs twice during the 2nd and 3rd d and 40% reported drug use only on the 2nd d. In CTRL G, one patient used drugs and was therefore excluded from the statistical analyses. - KLS G experienced significantly less pain during the 4th, 5th, and 6th d compared to P G. - KLS G and P G experienced significantly more pain during all d compared to CTRL G. - A total of 94% of patients reported at least some discomfort during RME activation. Pain location: 88.9% at anchoring tooth level, followed by frontal tooth (33.3%), nose (14.3%), and head (9.5%). Phase 2: - KLS-B G reported almost no pain during the whole RME activation. - Significantly less pain was reported for the first 3 d of activation, and good pain control was observed even during the other d. - Analgesics taken during the first 3 d seem to be even more effective at reducing pain from the 1st d of activation, with almost no pain for the whole activation period. - KLS is an interesting and valid option in this particular setting, thanks to its efficacy and mechanism of action.	- Phase 1: randomized controlled clinical trial. - Phase 2: prospective case series.
TBE G with TBBE G.	- Patient pain and discomfort during RME placement was overall low and did not differ significantly between the two groups. The main complaint was pressure during RME application. - No significant differences in pain levels between groups were observed, although patients in TBE G generally scored higher. - No significant differences in pain levels were observed between d 1 and 4 in TBE G. TBBE G scored significantly lower for pain in the molars and incisors on the 4th d compared to the 1st d. - Pain levels in the jaw, palate, and tongue were minor and did not differ significantly within/between groups. - No significant differences in discomfort were observed between groups. No significant differences in discomfort were observed between d 1 and d 4 in TBE G, but TBBE G scored significantly lower jaw tension. - Age positively correlated with overall pain and discomfort on the 4th d of treatment. - Very few sex differences were observed, though F complained more about tension from teeth than M. - Analgesic consumption was low and did not differ significantly within/between groups. Paracetamol and Ibuprofen were the most commonly used.	Randomized controlled trial.
None.	- No significant differences were observed between rate of expansion and gender, age, or dentition stage. - A total of 98% of patients reported some pain during RME. Maximum reported pain occurred during the first six turns, then reported pain steadily and significantly decreased with time. - No statistically significant difference was observed between the two sexes in median pain scores. No difference was observed in reported pain based on age or sex. - Patients whose expansion rate was 2 turns/d were 2.1 times more likely to report pain than patients whose expansion rate was 1 turn/d. Regarding the first 10 turns, patients whose expansion rate was 2 turns/d were 3.0 times more likely to report pain than patients whose expansion rate was 1 turn/d. No difference was observed in reported pain during the last 10 d of turns. - Pain medication (including Children’s Tylenol^®^, Advil^®^, Motrin^®^) was taken after 7% of turns, and 69% of the time pain medication was taken during the first 6 turns. A total of 48% of patients took medication at least one time during RME. - No difference was observed in pain medication use during the last 10 d of turns. No difference in pain medication use was observed based on age or sex.	Not defined by authors.

F = female/s, M = male/s, m.a. = mean age, G = group, RME = rapid palatal expansion/expander, LE = Leaf expander, min = minutes, h = hour/s, d = day/s, w = week/s, m = month/s, pt = point/s, NRS = Numerical Rating Scale, FPS = Faces Pain Scale, FPS-R = Faces Pain Scale-Revised, GRS = Graphic Rating Scale, CAS = Color Analog Scale, PBMT = photobiomodulation therapy, TBE = tooth-borne expander, BBE = bone-borne expander, TBBE = Tooth–bone-borne expander, KLS = ketoprofen lysine salt, P = paracetamol/acetaminophen, CTR = control.

**Table 4:** Methodological Quality of included studies.

Authors	Items for Methodological Quality Criteria											Total Score	Methodological Quality of the Study
	A	B	C	D	E	F	G	H	I	J	K		
Caccianiga et al., 2022 [2]	1	1	0	0.5	1	0.5	1	1	1	1	1	9	Moderate (7–9 points)
de Araújo et al., 2021 [3]	0.5	1	1	0.5	1	0	1	1	1	0.5	1	8.5	Moderate (7–9 points)
Matos et al., 2021 [15]	1	1	1	0.5	1	0.5	1	1	1	1	1	10	Good (>9 points)
Nieri et al., 2021 [16]	1	1	1	1	1	0.5	1	1	1	1	1	10.5	Good (>9 points)
Altieri et al., 2020 [1]	0.5	1	0	0.5	1	0.5	1	1	1	0.5	1	8	Moderate (7–9 points)
Ugolini et al., 2019 [12]	1	1	1	1	1	0.5	1	1	1	1	1	10.5	Good (>9 points)
Cesur et al., 2018 [5]	0	1	0	0.5	1	0	0	1	1	0.5	1	6	Poor (<7)
Cossellu et al., 2018 [9]	1	1	1	0.5	1	0.5	1	1	1	1	1	10	Good (>9 points)
Feldmann et al., 2017 [17]	1	1	0	0.5	1	0.5	1	1	1	0.5	1	8.5	Moderate (7–9 points)
Needleman et al., 2000 [10]	0	1	0	0.5	1	0	0	1	1	0.5	1	6	Poor (<7)
